# Cardiovascular risk prevention awareness and practices in type 2 diabetes: Linking HbA1c and lipid levels

**DOI:** 10.6026/973206300222129

**Published:** 2026-04-30

**Authors:** Nandita Tallam, Harshitha Kumar, Sneha Parve, Kiran Tawalare, Aswathy T Menon, Pranathi Danda, Gourav Singh Shekhawat

**Affiliations:** 1 Department of Anatomy, Mediciti institute of Medical Sciences, Telangana India; 2 Department of OHC, UR Life Apollo wellness, Karnataka, India; 3 Department of Community Medicine, NKP Salve Institute of Medical Sciences & RC and Lata Mangeshkar Hospital, Nagpur, India; 4 Department of Ayurveda, Shri Ayurved College, Nagpur, India; 5 Department of General Medicine, Sree Balaji Medical College, Tamil Nadu, India; 6 Department of Internal Medicine, Mediciti Institute of Medical Sciences, Medchal, Hyderabad, Telangana, India; 7 Department of Community Medicine, Rajasthan University of Health Science and Jai Durga College of Nursing, Rajasthan India

**Keywords:** Diabetes mellitus, cardiovascular risk prevention, knowledge-attitude-practice, lipids, HbA1c

## Abstract

Cardiovascular risk remains a leading cause of morbidity and mortality in patients with type 2 diabetes mellitus (T2DM), yet awareness and preventive practices are often inadequate. Therefore, it is of interest to evaluate cardiovascular risk prevention awareness and practices among T2DM patients and examined their correlation with HbA1c and lipid profiles. Data on lifestyle practices, medication adherence and risk awareness were collected and analyzed alongside glycemic and lipid parameters. Higher awareness and better preventive practices were significantly associated with improved glycemic control and favorable lipid levels. Thus, we show the importance of targeted patient education to enhance cardiovascular risk prevention and optimize metabolic outcomes in T2DM.

## Background:

Type 2 diabetes increases the chances of developing a heart disease and is among the reasons people die prematurely [1, 2]. Recent findings suggest that people with diabetes need to keep their blood sugar, cholesterol, blood pressure and lifestyle in check to have lower rates of cardiovascular disease [3, 4]. Many people with diabetes do not reach their target HbA1c or LDL cholesterol levels [5, 6]. Knowledge of the risks associated with heart disease and treatment options impacts on patients' ability to take their medications and to make long-term changes in their behaviour. However, knowledge has not effectively translated into behaviour change to prevent heart disease [7, 8]. Recent studies demonstrate that behaviours (diet, exercise, compliance with medications) have greater impact on patients' diabetes control than knowledge alone [9, 10]. This makes it clear that there are cultural barriers to putting into practice the knowledge gained by diabetic patients about caring for their diabetes. Very few studies have looked at the relationships between knowledge-attitude-behaviour (KAB) domains and laboratory measurements of metabolic control (HbA1c, LDL-C, non-HDL-C) using the same cohort of patients [11, 12-13]. Therefore, it is of interest to measure whether or not diabetic adults are aware of the cardiovascular risks of their diabetes and what habits they have to reduce their cardiovascular risk and whether or not their behaviours are related to their glycaemic and lipid levels.

## Materials and Methods:

In this six-month cross-sectional research conducted in a tertiary care hospital's outpatient diabetes clinic, 200 adults ages 30-75 years with a diagnosis of type 2 diabetes for at least one year were consecutively recruited with their first written informed consent. Enrolment criteria excluded participants who had secondary diabetes, were within three months of suffering an acute cardiovascular event, had active infections, or had cognitive impairment. Institutional ethics board approval was obtained prior to study onset. A validated cardiovascular risk prevention Knowledge, Attitude and Practice (KAP) questionnaire was administered by trained staff. This instrument contained a total of 30 items related to knowledge (12), attitude (8) and practice (10) regarding lipid levels and HbA1c goals; dietary habits; physical activities; smoking status; and adherence to statin or anti-platelet drugs. Each correct knowledge response or an attitude considered positive was counted as one point for the KAP score. Participants were placed into three groups (i.e., good, average, poor) based on how they compared to other participants in their respective tertiles across the KAP score for a given domain(s). Following the KAP Questionnaire, venous blood was collected to determine HbA1c and fasting lipid (LDL-C; non-HDL-C; triglycerides) profile levels. Clinical records provided demographic information, the duration of diabetes, additional disease states, body mass index (BMI) and drug therapies. The statistical software used to conduct the analysis was SPSS version 26. Continuous variables were expressed as (mean ± standard deviation) and categorical variables as frequency (%). Relationships between KAP scores and laboratory values were examined using Pearson's correlation coefficient; and multivariable linear regression models adjusted for age, gender, duration of diabetes, BMI and statin use. A statistically significant result was deemed as a p-value less than 0.05 for all statistical tests conducted in this study.

## Results:

A total of 200 adults with type 2 diabetes were included in the analysis. The mean age was 58.7 ± 8.4 years and 55% were male. The mean duration of diabetes was 9.3 ± 5.2 years and mean BMI was 26.4 ± 3.7 kg/m². Hypertension was present in 60%, dyslipidaemia in 54% and 75% were receiving statin therapy. The mean knowledge score was 7.8 ± 2.4 (out of 12), mean attitude score was 6.4 ± 1.7 (out of 8) and mean practice score was 6.9 ± 2.2 (out of 10). The mean HbA1c was 8.1 ± 1.6%, mean LDL-C was 110 ± 28 mg/dL and mean non-HDL-C was 141 ± 34 mg/dL. Practice score demonstrated significant negative correlation with HbA1c (r = -0.42, p < 0.001) and LDL-C (r = -0.35, p < 0.01). Knowledge score showed weaker correlations, while attitude score demonstrated modest associations. In multivariable regression, practice score remained an independent predictor of HbA1c and LDL-C after adjustment for confounders. Specific preventive behaviours such as regular physical activity and consistent statin use were associated with better biochemical control. Table 1 (see PDF) shows that the mean age was 58.7 ± 8.4 years, 55% were male, mean diabetes duration was 9.3 ± 5.2 years and 60% had hypertension while 75% were receiving statin therapy. Table 2 (see PDF) indicates that the mean knowledge score was 7.8 ± 2.4 with only 16% classified as good knowledge, while 29% demonstrated good preventive practice and 15% had poor practice levels. Table 3 (see PDF) demonstrates that HbA1c decreased from 8.7% in the low-practice group to 7.4% in the high- practice group and LDL-C declined from 122 mg/dL to 98 mg/dL across tertiles, with statistically significant differences. Table 4 (see PDF) shows that practice score had the strongest negative correlation with HbA1c (r = -0.42) and LDL-C (r = -0.35), whereas knowledge and attitude scores demonstrated weaker associations. Table 5 (see PDF) highlights that practice score independently predicted lower HbA1c (β = -0.28, p = 0.003) and LDL-C (β = -0.25, p = 0.006) after adjustment, while statin use independently predicted lower LDL-C levels. Table 6 (see PDF) compares achievement of biochemical targets and shows that consistent statin use was associated with the highest proportion of LDL-C control (72%) and regular physical activity was associated with improved HbA1c control (62%). Table 7 (see PDF) depicts that the most frequently reported barriers were lack of awareness of cholesterol targets (42%), time constraints for exercise (36%) and perceived difficulty in lifestyle change (34%).

## Discussion:

It has been shown through this research that for patients with type 2 diabetes preventive behaviour has a stronger correlation with glucose control and cholesterol levels when compared to knowledge alone or attitude alone. The study demonstrates that a positive behaviour score for practice of preventive behaviour represents a moderately negative correlation with HbA1c (r = -0.42) and LDL-C (r = -0.35), whereas the knowledge component had a much lower association than either practice or attitude [14]. Additionally, when evaluated through multiple regression analysis, we found that preventive behaviours remained an independent predictor of HbA1c and LDL-C by controlling for age, gender, treatment and other comorbidities. Thus, this study provides evidence that behavioural engagement is a measurable and important independent contributor to metabolic risk [15]. Historically, we have been aware that cardiovascular risks associated with diabetes are influenced by both elevated blood glucose and elevated levels of cholesterol [1, 2]. Despite clinical practice guidelines recommending blood glucose (HbA1c) levels of <7% and total cholesterol levels below 130 mg/dL, many patients continue to have elevated HbA1c levels and elevated levels of LDL-C [3, 4]. In this cohort, the mean HbA1c level was 8.1% and the average level of LDL-C was 110 mg/dL, even though 75% of participants were prescribed statin therapy; thus, it is reasonable to conclude that medications alone may not be effective without ongoing behavioural support [16]. The gradient observed across the three practice terminals is of clinical significance. Those within the hugest practice category had HbA1c levels 1.3% lower and LDL-C levels 24 mg delayed than individuals within the lowest practice terminal. Studies suggest that even minor reductions (0.5%-1.0%) of HbA1c can lead to significant reductions in microvascular and cardiovascular risks [5]. Similarly, reductions of LDL-C of 20-30 mg/dL are linked to significant decreases in the incidence of coronary artery disease events due to increased levels of atherosclerotic progress [6]. Practical differences illustrate how the implementation of preventative behaviour has direct effects on an individual's risk of cardiovascular disease [17]. Knowledge is just a piece of the puzzle but does not necessarily mean that the person is able to act on it; therefore, even when the knowledge surface has been addressed, there is still a significant gap between what an individual knows and how they choose to live their life. Recent studies have shown that people must be made aware of what they can do to change their daily behaviours and even knowing about changing their lifestyle does not guarantee that they will make these changes [7, 8]. To translate knowledge into real-world behaviours such as exercising consistently, eating healthily and taking medication as prescribed requires both motivational support and structural assistance. Our results further solidify that an individual's metabolic control will depend on how well they actually execute their prescribed preventive behavioural practices, not just whether or not they are being informed about them [18]. Specific preventive behaviours were associated with significantly greater or lesser rates of achievement of targeted metabolic control. For example, sufficient amounts of physical activity and adherence to stated recommendations for statin therapy were associated with significantly higher rates of achievement of the targets set forth [19]. This supports other recent studies which show that regular engagement in physical activity increases insulin sensitivity and lipid metabolism, while adherence to statin therapy decreases variability in LDL-C levels and reduces the risk of developing cardiovascular disease [9, 10]. It is noteworthy that individuals' responses about the barriers they face to performing their prescribed behaviours indicate modifiable intervention points (e.g., lack of awareness of cholesterol target numbers, lack of time to exercise) [20]. The contribution of this study is that it assesses the Knowledge, Attitude and Practice (KAP) measures in conjunction with the biochemical measures of the subjects. This study was able to quantify the independent contributions of the preventive practices on the metabolic outcomes. Prior studies have either indicated the level of knowledge about preventive behaviour or have indicated that practice behaviour is an independent predictor of lipid and glycaemic control when controlling for confounding factors. Therefore, we anticipate that behavioural assessment will be used as a tool for assessing risk stratification related to diabetes management. Limitations to this study include the inability to make causative determinations due to the cross-sectional design. Preventive behaviours were self-reported and thus subject to recall bias. This single-centre study may also limit its generalisability. Further longitudinal and interventional research is warranted to determine if behavioural modifications improve individuals' practice scores and consequently, their overall cardiovascular risk. In conclusion, this study indicates that the integration of a standardised assessment of KAP-related behaviours into routine diabetes management is recommended. KAP domains represent modifiable components of an individual's cardiovascular risk and create an accessible avenue to improving metabolic outcomes beyond just medication use.

## Conclusion:

Preventive practice behaviour is independently associated with improved glycaemic and lipid control in adults with type 2 diabetes. Knowledge alone does not translate into metabolic benefit without consistent behavioural execution. Integrating structured behavioural assessment and targeted lifestyle interventions into routine diabetes care may strengthen cardiovascular risk reduction strategies.
